# SMRT sequencing of full-length transcriptome and gene expression analysis in two chemical types of *Pogostemon cablin* (Blanco) Benth.

**DOI:** 10.7717/peerj.12940

**Published:** 2022-02-22

**Authors:** Hongyi Zhang, Wenjing Deng, Changhua Lu, Mengling He, Hanjing Yan

**Affiliations:** College of Traditional Chinese Medicine, Guangdong Pharmaceutical University, Guangzhou, Guangdong, China

**Keywords:** *Pogostemon cablin* (Blanco) Benth., SMRT, Transcriptome, Two chemical types, Terpenoid biosynthesis

## Abstract

**Background:**

*Pogostemon cablin* (Blanco) Benth. also called patchouli, is a traditional medicinal and aromatic plant that grows mainly in Southeast Asia and China. In China, *P. cablin* is divided into two chemical types: the patchouliol-type and the pogostone-type. Patchouliol-type patchouli usually grow taller, with thicker stems and bigger leaves, and produce more aromatic oil.

**Methods:**

To better understand the genetic differences between the two chemical types that contribute to their differences in morphology and biosynthetic capabilities, we constructed *de novo* transcriptomes from both chemical types using the Pacific Biosciences (PacBio) Sequel platform and performed differential expression analysis of multiple tissues using Illumina short reads.

**Results:**

In this study, using single-molecule real-time (SMRT) long-read sequencing, we obtained 22.07 GB of clean data and 134,647 nonredundant transcripts from two chemical types. Additionally, we identified 126,576 open reading frames (ORFs), 100,638 coding sequences (CDSs), 4,106 long noncoding RNAs (lncRNAs) and 6,829 transcription factors (TFs) from two chemical types of *P. cablin*. We adopted PacBio and Illumina sequencing to identify differentially expressed transcripts (DEGs) in three tissues of the two chemical types. More DEGs were observed in comparisons of different tissues collected from the same chemical type relative to comparisons of the same tissue collected from different chemical types. Furthormore, using KEGG enrichment analysis of DEGs, we found that the most enriched biosynthetic pathways of secondary metabolites of the two chemical types were “terpenoid backbone biosynthesis”, “phenylpropanoid biosynthesis”, “plant hormone signal transduction”, “sesquiterpenoid and triterpenoid biosynthesis”, “ubiquinone and other terpenoid-quinone biosynthesis”, “flavonoid biosynthesis”, and “flavone and flavonol biosynthesis”. However, the main pathways of the patchouliol-type also included “diterpene biosynthesis” and “monoterpene biosynthesis”. Additionally, by comparing the expression levels of the three tissues verified by qRT-PCR, more DEGs in the roots were upregulated in the mevalonate (MVA) pathway in the cytoplasm, but more DEGs in the leaves were upregulated in the methylerythritol phosphate (MEP) pathway in the plastid, both of which are important pathways for terpenoids biosynthesis. These findings promote the study of further genome annotation and transcriptome research in *P. cablin*.

## Introduction

*Pogostemon cablin* (Blanco) Benth., called patchouli in English, is an economically important plant in the family Lamiaceae and is widely cultivated in the tropical and subtropical areas of Asia (Wu et al., 2010). In China, *P. cablin* is valued for its well-known medicinal properties and used to treat the common cold, headache, fever, vomiting, indigestion, and diarrhea (Chinese Pharmacopoeia Commission, 2020). A crucial essential oil (called patchouli oil) can be produced from patchouli leaf extract. There are more than 24 different sesquiterpenes in patchouli oil, such as patchouli alcohol, eugenol, α-bulnesene, rosmarinic acid, and others (Deguerry, Pastore & Wu, 2006; Hsu et al., 2006). More than 50% of patchouli oil is patchouli alcohol (Miyazawa et al., 2000). Patchouli volatile oil is an important ingredient for perfumes, incense, soaps, and other cosmetic products (Fan et al., 2011; Chen et al., 2013). In China, there are two chemical types, called patchouliol-type patchouli and pogostone-type patchouli. Patchouliol-type patchouli contains a patchouli alcohol content that is much higher than the pogostone content. If the patchouli alcohol content is equal to or less than the pogostone content in the stems and leaves, it is called pogostone-type patchouli (Luo et al., 2003). Patchouliol-type and pogostone-type patchouli also exhibit differences in chemical composition (Hu et al., 2019), morphology (Li et al., 2002) and genetics (Liu et al., 2021).

Patchouli alcohol, the major component in patchouli oil, is a sesquiterpene. Its biosynthesis includes terpenoid backbone biosynthesis and sesquiterpenoid biosynthesis pathways. The synthesis of plant terpenoids involves two main routes: the mevalonate (MVA) pathway and the methylerythritol phosphate (MEP) pathway, with the former located in the cytoplasm and the latter in plastids. Both pathways can synthesize isopentenyl pyrophosphate (IPP), which is the central precursor of the synthesis of all terpene compounds (Sallaud et al., 2009). The direct precursor of sesquiterpene, farnesyl diphosphate (FPP), is derived from IPP. Does the MVA pathway or MEP pathway play a more important role for sesquiterpene synthesis? The answer could be important for increasing the content of patchouli alcohol by the biometabolic engineering pathway.

In recent years most transcriptomes are generated on the Illumina platform using Next-generation sequencing (NGS) technology (Chen, Qiu & Yang, 2019). The first *de novo* assembled 1.15 Gb draft genome sequence for *P. cablin* were performed using NGS technology in 2016 (He et al., 2016). However, NGS sequencing reads are short, which makes it difficult to perform subsequent bioinformatics analyses, such as alternative splicing, assembly, and annotation (Tilgner et al., 2015; Koren et al., 2012). Thus, single-molecule real-time (SMRT) sequencing (Pacific Biosciences of California, Inc., Menlo Park, CA, USA) was developed, which overcomes the limitation of short-read sequences by enabling the generation of kilobase-sized sequencing reads (Jia et al., 2018). This makes the full-length transcriptome particularly important for basic and applied research on gene function, gene expression, and regulation. In 2019 the first full-length transcriptome of *P. cablin* were analyzed by using SMRT sequencing and 102 transcripts were annotated as 16 encoding enzymes involved in patchouli alcohol biosynthesis (Chen et al., 2019). With the help of highly accurate NGS short reads correction and circular consensus sequence (CCS) reads self-correction, SMRT sequencing achieves results with >99% accuracy (Qian et al., 2019). Therefore, sequencing approaches that combine SMRT and short-read sequencing for transcriptome assembly and differential expression analysis provides a highly accurate assembly that can be used for downstream evolutionary and gene function analysis.

In this experiment, we used SMRT sequencing and NGS to perform transcriptome sequencing of two chemical types, namely, the patchouliol-type and the pogostone-type. Based on the transcriptome data, we obtained transcript functional annotation, predicted open reading frames (ORFs), and analyzed alternative splicing (AS), transcription factors (TFs) and long noncoding RNAs (lncRNAs). In addition, the transcriptomes of the two chemical types will help to further understand the detailed biosynthesis mechanisms of patchouli alcohol and other terpenoids in *P. cablin*.

## Materials and Methods

### Plant materials and RNA preparation

The samples of *P. cablin* were cultivated in Sihui (N23°29′53″, E112°46′57″) and Gaoyao (N22°55′92″, E112°28′38″), Guangdong Province, China. The pogostone-type germplasm came from Shipai, Guangzhou City about 70 years ago and has been cultivated in Gaoyao. Nowadays the pogostone-type germplasm is very rare in the markets. The patchouliol-type germplasm from Sihui is a mainstream product in the markets and is large-scale cultivated in Guangdong and Hainan Province. Two chemical types were cultivated in Sihui and Gaoyao respectively and got the same field management such as weeding, watering and fertilizing in the period from transplanting to harvesting. Patchouli harvesting day was also the cutting breeding day. The apical stem cuttings of two germplasms were planted in Sihui and Gaoyao respectively on November 18, 2017. On March 15, 2018, the cutting seedlings were transplanted to the medicine garden of Guangdong Pharmaceutical University in Guangzhou on the same day. During the growth period, all the samples were given the same field management including weeding and watering. On November 7, 2018, the mature and healthy leaves and corresponding stems were collected in liquid nitrogen. The roots were cleaned up and also collected as samples. Three biological plant replicates per experimental treatment were harvested. All samples were immediately frozen in liquid nitrogen and stored in a −80 °C freezer until RNA isolation. The samples from Sihui belong to the patchouliol-type, while the samples from Gaoyao belong to the pogostone-type. No specific permits were required and no protected species were involved in this study.

Total RNA samples were isolated from each sample using an RNAprep Pure Plant kit (TIANGEN Biotech, Beijing, China) and treated with DNase I to remove DNA contaminants (TIANGEN) according to the manufacturer’s instructions. The RNA quantity and quality were assessed using the RNA Nano 6000 Assay Kit of the Agilent Bioanalyzer 2100 system (Agilent Technologies, Santa Clara, CA, USA). RNA concentration was measured using a Qubit® RNA Assay Kit in a Qubit® 2.0 Flurometer (Life Technologies, Carlsbad, CA, USA).

### Library preparation and SMRT sequencing

The RNAs of three tissue samples of the two chemical types, including roots, stems and leaves, were pooled at equal ratios, respectively. The SMART sequencing library was prepared according to the Iso-Seq protocol as described by Pacific Biosciences. Full length cDNAs were synthesized using the SMRTer cDNA synthesis kit and fragments of 1–6 kb in size were collected using a BluePippin instrument. Two cells (F01 and F02) were run on the PacBio RSII platform. The resulting library was sequenced using the Iso-Seq function of the PacBio RS II system (Pacific Biosciences, Menlo Park, CA, USA) (Wang et al., 2019).

### Illumina RNA-seq library construction and sequencing

Total RNA from 18 samples of three tissues of the two chemical types (three biological replicates with each tissue) was used to construct RNA-seq libraries. First, mRNA was interrupted to short fragments after fragmentation buffer was added. Then the first strand cDNA was synthesized with the resultant mRNA fragments as templates. The second strand cDNA was synthesized by using RNase H and DNA polymerase I. After end repair and A-tailing, the purified double-stranded cDNA fragments were ligated with paired-end adaptors, and then used AMPure XP beads to select fragments size larger than 100 bp. Then the fragments of appropriate size were amplified by PCR to obtain the final cDNA libraries (Dong et al., 2015). The quality of the libraries was assessed on the Agilent 2100 Bioanalyzer (Agilent Technologies, Santa Clara, CA, USA). Finally, the 18 libraries were sequenced using the Illumina HiSeq™ 2500 system (Illumina, San Diego, CA, USA) (Deng et al., 2018).

### PacBio read error correction

Raw SMRT sequencing reads were processed by removing read fragments whose lengths were less than 50 bp and accuracy was less than 80% to retain only the highest quality reads for downstream analysis. The reads of insert (ROIs) with both the 5′ and 3′ primer sequences and a poly(A) tail present were considered to be full-length transcripts. Chimeric sequences were identified and removed using UCHIME v4.2.40 (Edgar et al., 2011). The non-chimeric sequences in the full-length sequence are called full-length non-chimeric (FLNC) sequences. The iterative clustering for error correction (ICE) algorithm was used to obtain consensus sequences for isoforms. Sequences were then error corrected using Quiver v1 (Chin et al., 2013). The Proovread software v2.13.841 (Hackl et al., 2014) corrected the low-quality full-length transcript with its corresponding Ilumina RNA seq data. The 5′ terminal regions of transcripts may be degraded, causing reads derived from the same isoform to be divided into different clusters, leading to the occurrence of redundant sequences in the dataset. Therefore, CD-HIT (parameters: -c 0.95 -aS 0.99) was used to reduce redundancy among the FL transcripts. Finally, BUSCO v3.0.2 analysis was performed using embryophyta_odb10 dataset (Simão et al., 2015) to evaluate the integrity of the dereplicated transcriptome. The PacBio SMRT raw reads and the Illumina short reads have been submitted to the BioProject database of the National Center for Biotechnology Information have been submitted to the BioProject database of National Center for Biotechnology Information (accession numbers PRJNA606892 and PRJNA607886, respectively).

### Identification of ORFs, TFs and lncRNAs

TransDecoder v5.5.0 (Haas et al., 2013) was used to identify candidate protein coding regions with default parameters. ORFs of 200 nt in length or greater and those that had detectable Pfam (protein family) (Finn et al., 2014) domains (database downloaded November 2018) were retained for further analysis.

The TFs and transcript regulators (TRs) were predicted with iTAK software (Zheng et al., 2016) from putative protein sequences.

LncRNAs are an important component of the transcriptome. The most widely used methods for analyzing lncRNAs are CPC (Coding Potential Calculator) (Kong et al., 2007), CNCI (Coding-Non-Coding Index) (Sun et al., 2013), CPAT (Coding Potential Assessment Tool) (Wang et al., 2013) and Pfam protein structure domain analysis. Putative protein-coding RNAs were removed from the dataset. Transcripts of >200 nt in length predicted to contain more than two exons were considered candidate lncRNAs and were further screened using CPC/CNCI/CPAT/Pfam. HMMscan searches were first used to identify transcripts that contained pfam motifs. Transcripts that had coding potential, but did not have detectable pfam motifs, were considered potential lncRNAs and were further analyzed using CPC, CNCI, and CPAT. In this study, the results of all four analyses had to agree in order for a transcript to be annotated as a potential lncRNAs.

### Functional annotation of transcripts

Functional annotation of transcripts was performed using BLAST (version 2.2.26) (Altschul et al., 1997) to compare trascripts againt the NCBI nonredundant protein sequences (NR) (Deng et al., 2006), Swissprot (Bairoch & Boeckmann, 1991), Gene Ontology (GO) (Ashburner et al., 2000), Clusters of Orthologous Groups of proteins (COG) (Tatusov et al., 2000), euKaryotic Ortholog Groups (KOG) (Koonin et al., 2004), Pfam evolutionary genealogy of genes: Nonsupervised Orthologous Groups (eggNOG) (Jensen et al., 2008), Kyoto Encyclopedia of Genes and Genomes (KEGG) (Ogata et al., 1999) databases with an E-value threshold of 10^−5^. The alignments against the NR database were used in Blast2GO to obtain GO annotations. The KEGG pathway assignments were performed using KOBAS software (Mao et al., 2005).

### Screening of differentially expressed transcripts

The clean reads from Illumina RNA-Seq for 18 samples (three biological replicates of roots, stems, leaves of the pogostone-type and the patchouliol-type respectively) were separately mapped to the non-redundant SMRT reference by using Bowtie2 v2.3.4.2 (Langmead & Salzberg, 2012). Quantifications of the transcript expression values were determined by RSEM software (Li & Dewey, 2011) and then normalized to FPKM (fragments per kilobase of transcript per million mapped reads). The DESeq R package (1.10.1) was used to analyze gene expression differences between groups to identify differentially expressed genes (DEGs) (Anders & Huber, 2010). The resulting p-values were adjusted using Benjamini & Hochberg’s method to control the false discovery rate (FDR). DEGs were defined as by parameters of FDR < 0.01 and the absolute value of the log2 ratio ≥ 1. The Venn diagrams of DEGs in three tissues of two chemical types was illustrated using the online Venn diagram production website jvenn (http://jvenn.toulouse.inra.fr/app/index.html).

### Quantitative real-time PCR (qRT-PCR)

To validate transcript abundance as reflected by FPKM values for the assembled transcripts, qRT-PCR was performed to estimate relative expression levels with a SYBR® Premix Pro Taq HS qPCR Kit (Accurate Biotechnology Company, Guntur, Andhra Pradesh) on a CFX96 Real-Time PCR system (Bio-Rad, Hercules, CA, USA). Gene-specific primers were designed with NCBI and are described in File S1. The conditions for qRT-PCR amplification were as follows: incubation at 95 °C for 30 s, 40 cycles of 95 °C for 5 s and 60 °C for 30 s. The specificity of the primer amplicons was tested using melting curve analysis, and PCR products were verified by sequencing (Chen et al., 2019). The relative expression levels of the selected genes were normalized to the selected reference gene 18S rRNA and determined by the 2^−ΔΔCt^ method. The amplification contained three technical replicates and three biological replicates. The GraphPad Prism 8 software was used to analyze statistical significance. To analyze variance between the gene expressions in the different tissues of samples, one-way ANOVA (with Tukey *post hoc*) was used. A level of *p* < 0.05 was considered statistically significant.

## Results

### SMRT sequencing data output

Based on PacBio SMRT sequencing technology, clean reads of 11.31 Gb and 10.76 Gb were obtained from the two cells (F01: pogostone-type patchouli; F02: patchouliol-type patchouli). With a full pass ≥0 and quality >0.80, 574,817 and 420,978 ROIs were obtained, respectively (Table 1). In addition, 334,683 and 276,811 FLNC (full-length nonchimeric) read sequences were identified, respectively (Table 2).

**Table 1:** Reads of insert (ROI) statistics.

	F01	F02
cDNA size	1–6K	1–6K
Reads of insert	574,817	420,978
Read bases of insert	1,128,659,746	840,369,967
Mean read length of insert	1,963	1,996
Mean read quality of insert	0.93	0.94
Mean number of passes	9	11

**Table 2:** Full-length sequences statistics.

	F01	F02
Reads of insert	574,817	420,978
Number of five prime reads	393,474	322,245
Number of three prime reads	408,234	328,063
Number of poly-A reads	404,257	324,699
Number of filtered short reads	21,001	13,952
Number of non-full-length reads	215,392	126,856
Number of full-length reads	338,424	280,170
Number of full-length non-chimeric reads	334,683	276,811
Average full-length non-chimeric read length	1,846	1,965

### Transcript clustering analysis

A total of 169,641,152,493 consensus isoforms were obtained from F01 and F02, including 95,298 and 92,051 high-quality isoforms and 74,216 and 60,333 low-quality isoforms, respectively. The ICE clustering results were shown in Table 3. Dereplication analysis of high-quality transcripts and corrected transcripts was performed using CD-HIT to obtain 134,647 transcript sequences.

**Table 3:** Results of iterative clustering for error correction (ICE) clustering analysis.

	F01	F02
Number of consensus isoforms	169,641	152,493
Average consensus isoforms read length	2,028	2,147
Number of polished high-quality isoforms	95,298	92,051
Number of polished low-quality isoforms	74,216	60,333
Percent of polished high-quality isoforms (%)	56.18%	60.36%

### Prediction of coding sequences

**Figure 1:**
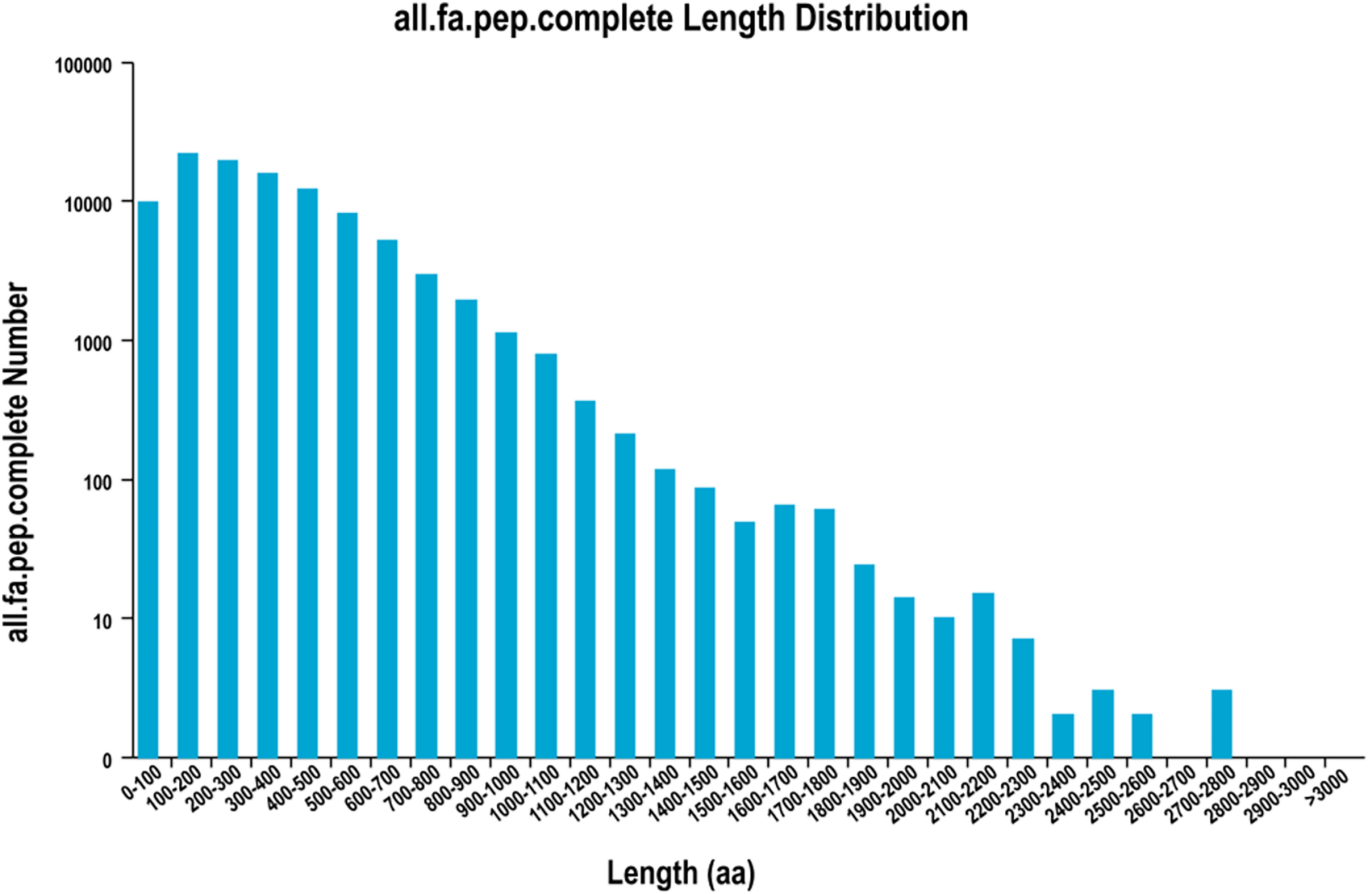
The distribution of the coding sequence lengths of the complete open reading frames.

A total of 126,576 ORFs were identified by using TransDecoder, including 100,638 complete ORFs. The distribution of the coding sequence length of the complete ORF is shown in Fig. 1.

#### Functional annotation of transcripts

A total of 52,511 transcripts were annotated in the COG; 75,289 annotations in the GO database; 55,168 in the KEGG database; 80,507 in the KOG database; 104,918 in the Pfam database; 94,944 in the Swiss-Prot database; 123,200 in the eggNOG database; and 126,527 in the NR database. In addition, a total of 128,264 transcripts were annotated in the eight databases (Table 4 and File S2).

**Table 4:** Annotation of transcripts using public databases.

Annotated databases	Isoform Number	Percentage
COG	52,511	40.94%
GO	75,289	58.70%
KEGG	55,168	43.01%
KOG	80,507	62.77%
Pfam	104,918	81.80%
Swiss-Prot	94,944	74.02%
eggNOG	123,200	96.05%
NR	126,527	98.65%
All	128,264	100%

### GO annotation

**Figure 2:**
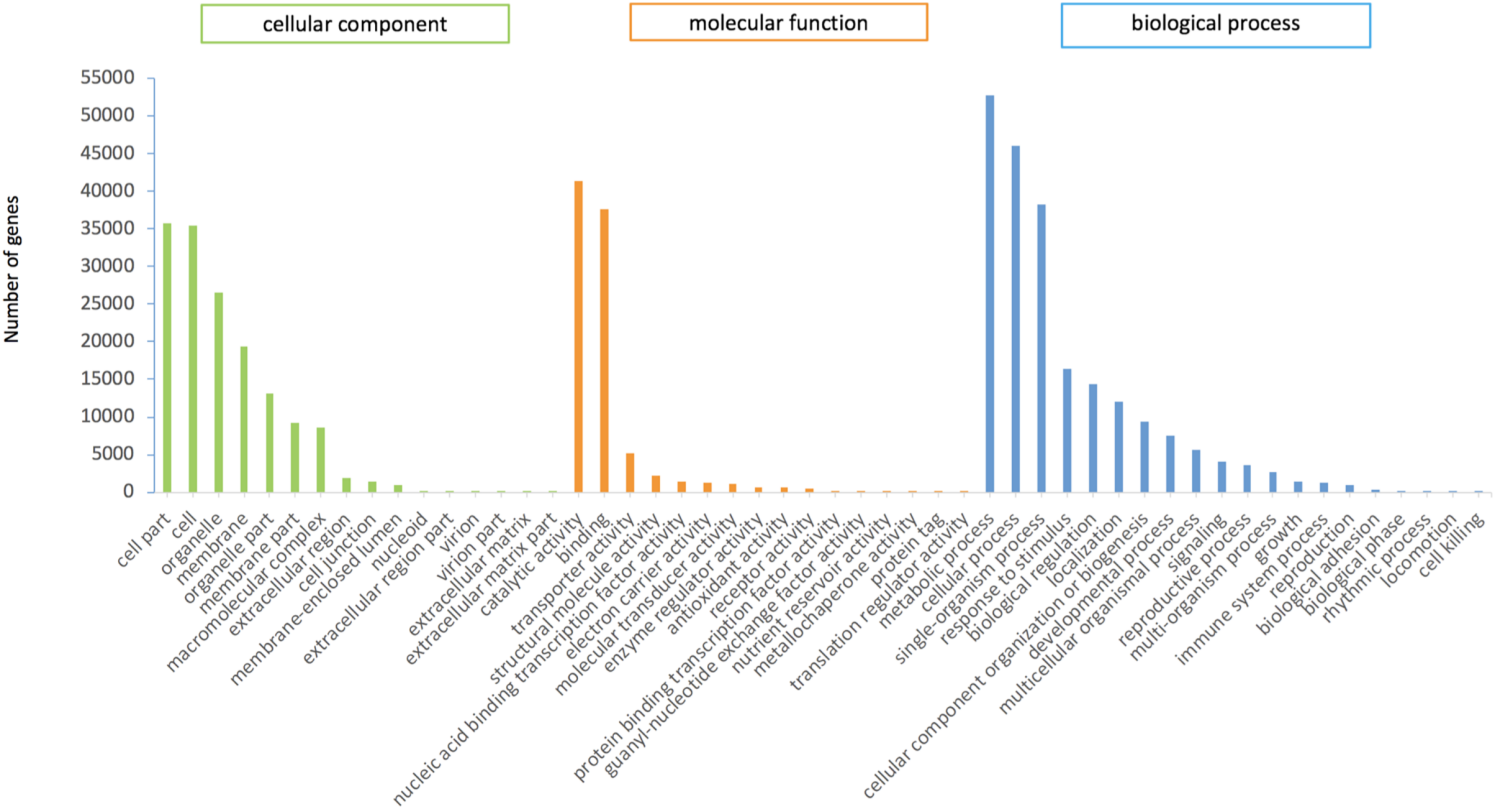
GO functional annotation of *P.cablin* transcripts. The x-axis represents GO categories; the y-axis represents the number of genes.

In this study, 75,289 transcripts were assigned GO terms, which were divided into three main categories. “Cell fraction” (GO: 0004464) and “cell” (GO: 0005623) dominated in the cellular component category. A large number of genes belonged to the molecular catalytic category of “catalytic activity” (GO: 0003824) and “binding” (GO: 0005488). The main subgroups of biological process category were “metabolic process” (GO: 0008152) and “cellular process” (GO: 0009987) (Fig. 2).

### KEGG annotation

KEGG pathway mapping of transcriptome assembly resulted in transcripts assigned to KEGG pathways. We found 1,695 transcripts related to terpenoid biosynthesis (Table 5 and File S3). Among them, 139 were annotated in the sesquiterpene and triterpene biosynthesis pathways, and 566 were annotated in the terpene skeleton biosynthesis pathway.

**Table 5:** The transcripts related to terpenoid biosynthesis.

Secondary metabolites biosynthesis pathways	Transcript numbers	Pathway ID
Terpenoid backbone biosynthesis	566	ko00900
Monoterpenoid biosynthesis	44	ko00902
Diterpenoid biosynthesis	103	ko00904
Sesquiterpenoid and triterpenoid biosynthesis	139	ko00909
Ubiquinone and other terpenoid-quinone biosynthesis	234	ko00130
Porphyrin and chlorophyll metabolism	319	ko00860
Limonene and pinene degradation	98	ko00903
Steroid biosynthesis	192	ko00100
Total	1695	

### lncRNA prediction

**Figure 3:**
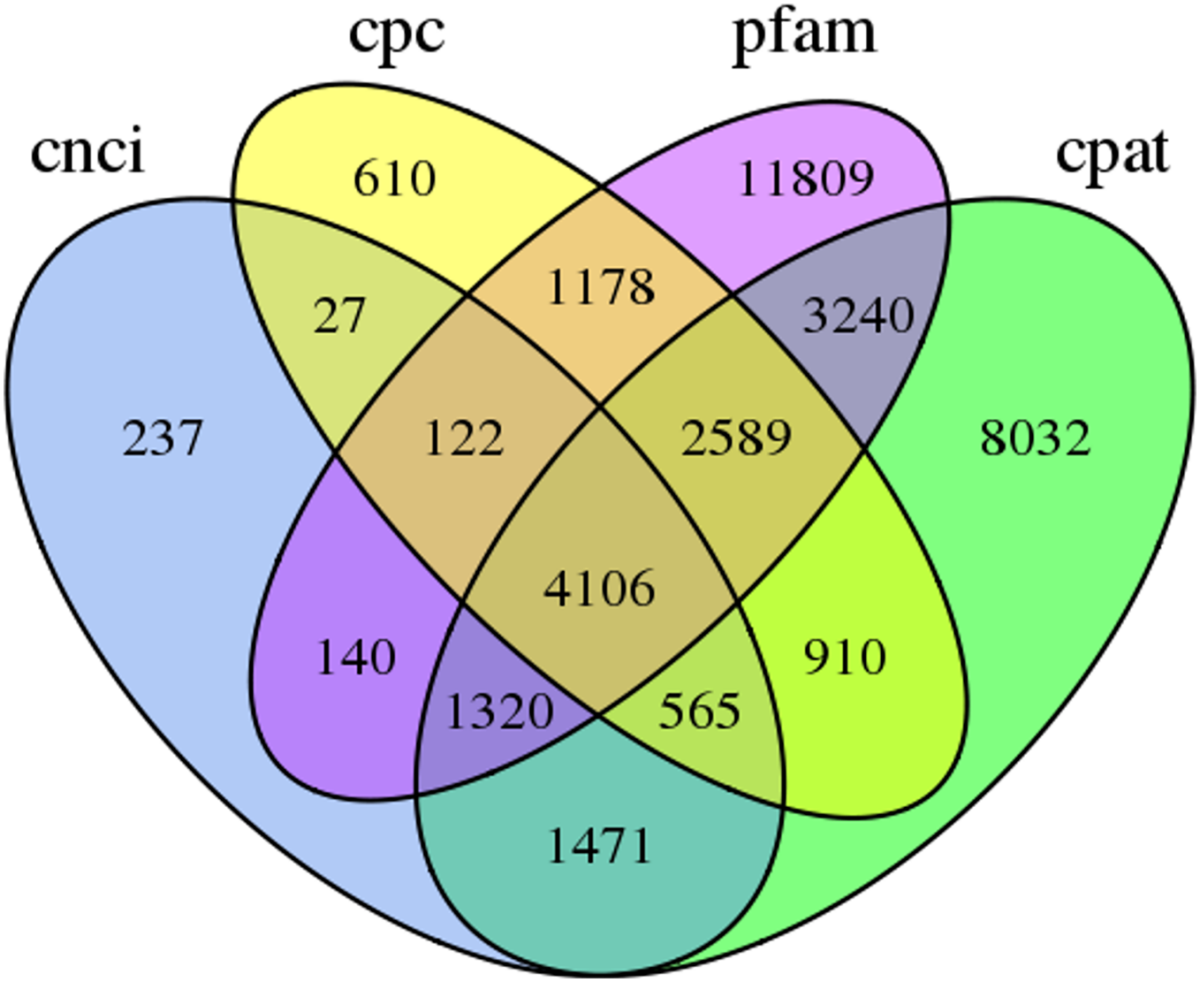
Venn diagram of the number of lncRNAs predicted by CPC, CNCI, CPAT and pfam protein structure domain analysis.

In this study, 4,106 lncRNA transcripts were predicted by CNCI, CPC, CPAT and Pfam (File S4). The length of lncRNAs varied from 280 bp to 8,941 bp, with 84.46% of lncRNAs having a length ≤2,000 bp. The mean length was 1,084 bp (Fig. 3).

### Analysis of TFs

**Figure 4:**
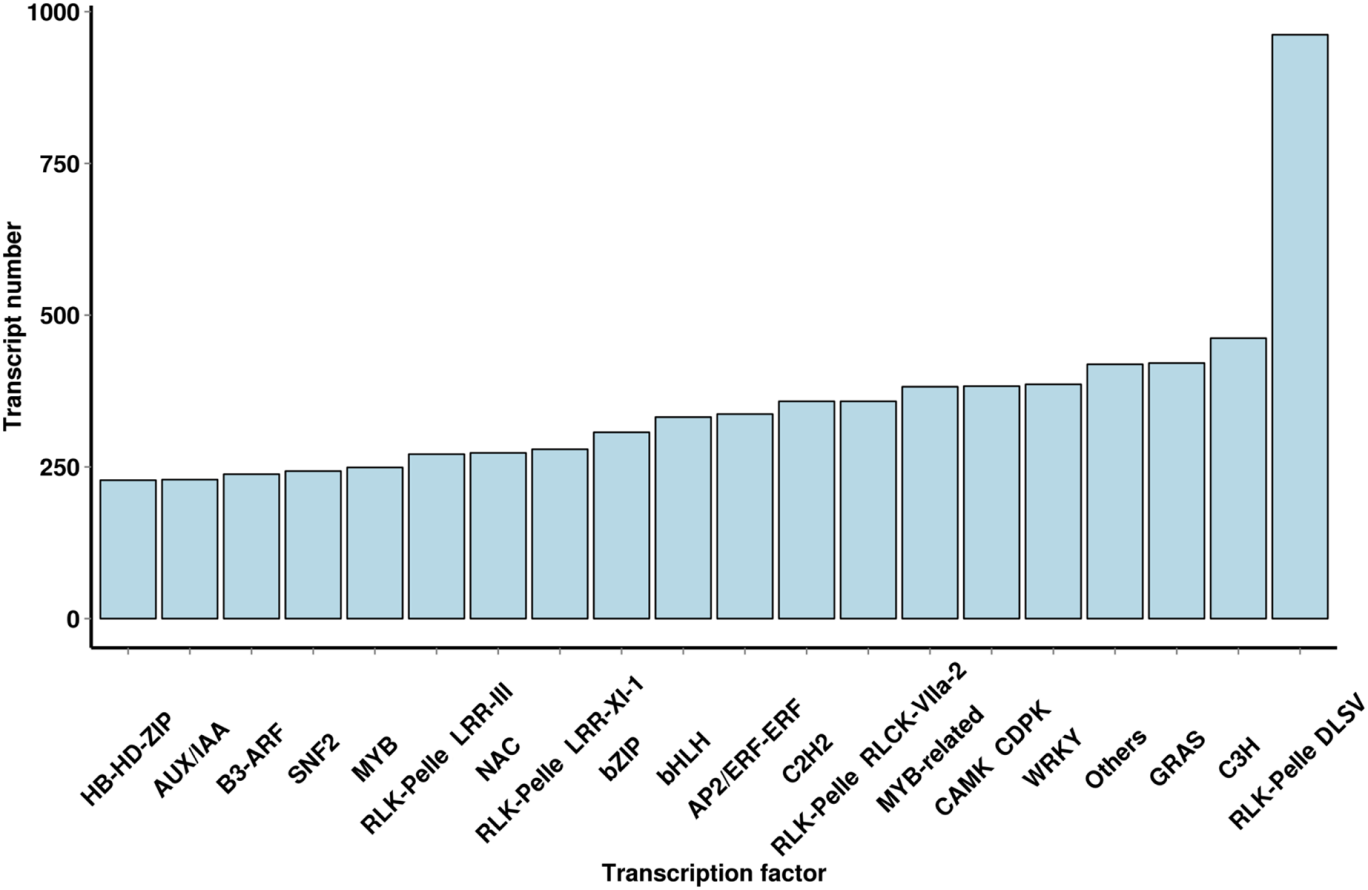
Number and family of top 20 TFs predicted by SMRT.

Using iTAK software, 6,829 TFs and 2,439 TRs were predicted; they belonged to 91 families of TFs, and a large number of TF families were found (Fig. 4 and File S5). The most abundant TF family was the WRKY (386 transcripts) family, followed by the AP2/ERF-ERF (337 transcripts), bHLH (332 transcripts), NAC (273 transcripts), MYB (249 transcripts) and AUX/IAA (229 transcripts) families. The TF family members provided additional mechanistic insights into the regulation of secondary metabolites.

### Analysis of differentially expressed genes (DEGs)

**Figure 5:**
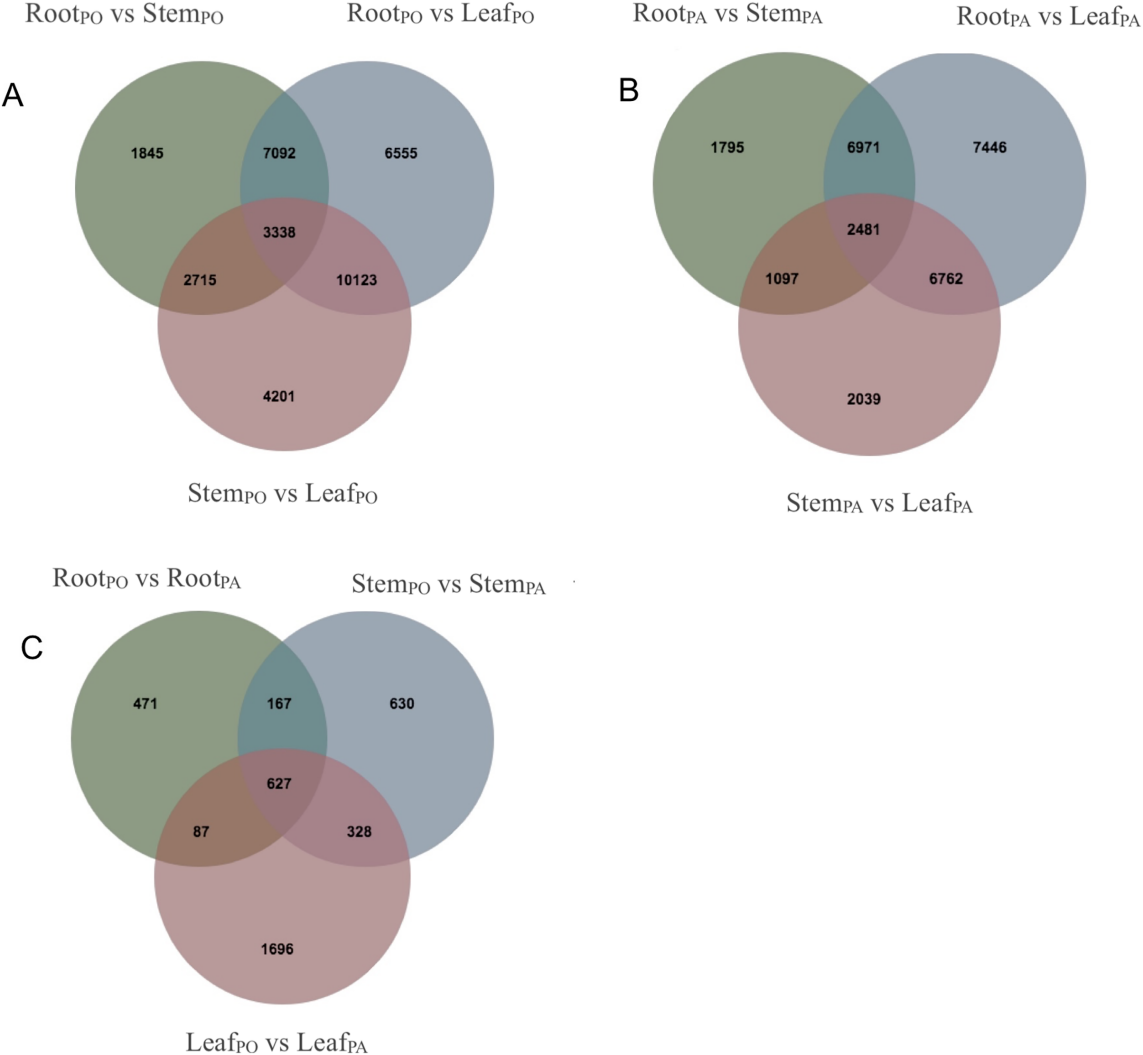
DEGs identified in comparisons among different tissues of *P.cablin*. (A) Venn diagram showing common and unique DEGs among different comparisons about the tissues of the pogostone-type (PO). (B) Venn diagram showing common and unique DEGs among different comparisons about the tissues of the patchouliol-type (PA). (C) Venn diagram showing common and unique DEGs among different comparisons about the tissues of two chemical types.

We analyzed DEGs of the roots, stems and leaves in the pogostone-type vs the patchouliol-type patchouli and found 5,842 DEGs (Files S6 and S7). A Venn diagram was made to visualize the numbers of overlapping and unique DEGs (Figs. 5A–5C). A total of 2,738 DEGs were in the leaves of the two chemical types, of which 1,833 were upregulated and 905 were downregulated. There were 1,752 DEGs in the stems of the two chemical types, of which 1,072 were upregulated and 680 were downregulated. There were 1,352 DEGs in the roots of the two chemical types, with 851 upregulated and 501 downregulated. More DEGs were mainly upregulated in the patchouliol-type than in the pogostone-type. For the same chemical type of patchouli, we compared the DEGs of three tissues. The DEGs in the roots vs leaves (23,660 DEGs in the patchouliol-type and 27,108 DEGs in the pogostone-type respectively) were much more abundant than in the roots *vs* stems (12,344 DEGs in the patchouliol-type and 14,990 DEGs in the pogostone-type respectively) and in the stems *vs* leaves (12,379 DEGs in the patchouliol-type and 20,377 DEGs in the pogostone-type respectively) (Figs. 5A and 5B).

### KEGG enrichment analysis of DEGs

**Figure 6:**
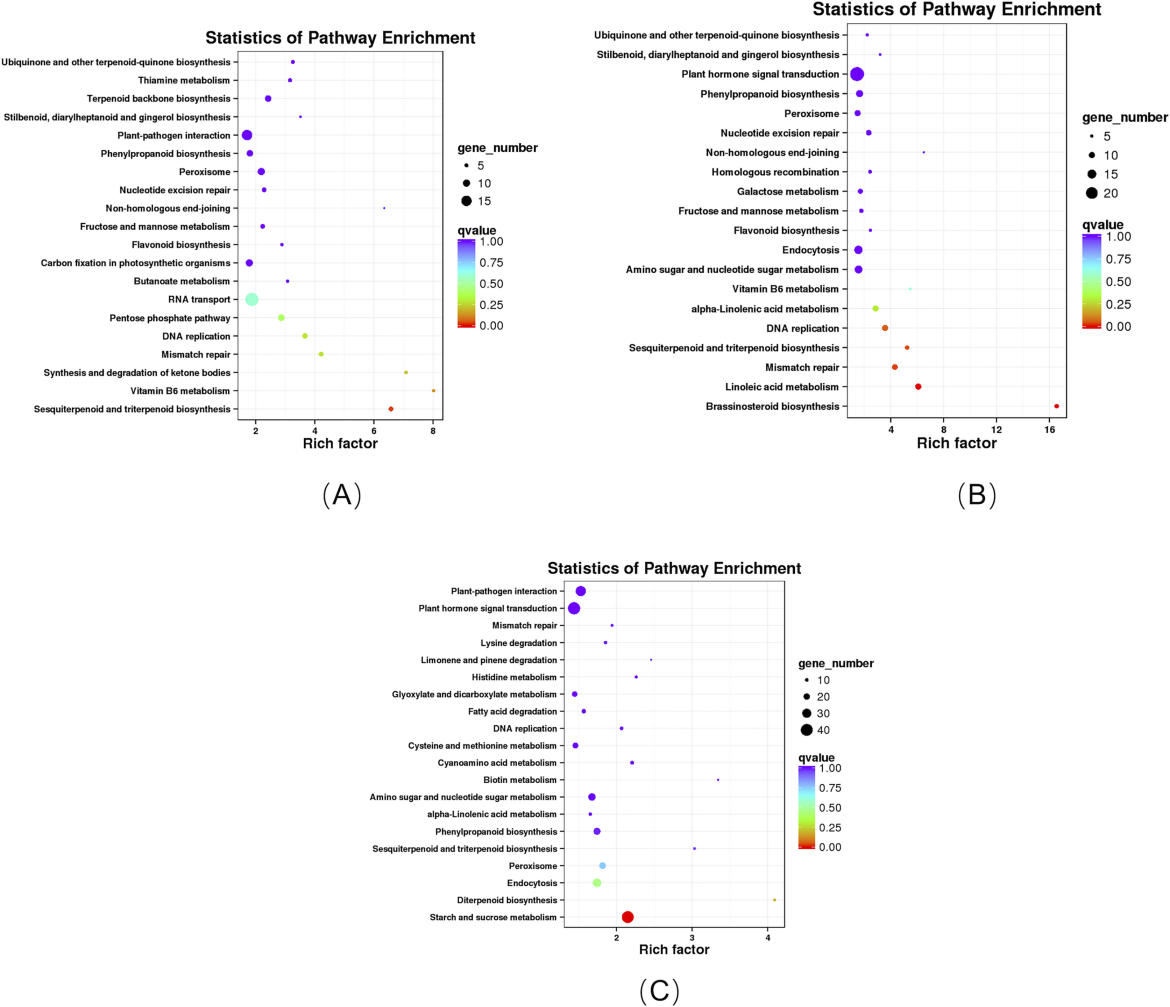
KEGG enrichment analysis of DEGs. (A) KEGG enrichment analysis of DEGs between the root of the pogostone-type vs the root of the patchouliol-type. (B) KEGG enrichment analysis of DEGs between the stem of the pogostone-type vs the stem of the patchouliol-type. (C) KEGG enrichment analysis of DEGs between the leaf of the pogostone-type vs the leaf of the patchouliol-type.

KEGG enrichment analysis revealed that these DEGs in the roots of the pogostone-type *vs* the patchouliol-type were significantly enriched in “sesquiterpenoid and triterpenoid biosynthesis” with q value ≤0.02815124 (four genes up regulated and two down regulated), “ubiquinone and other terpenoid-quinone biosynthesis” (five genes up regulated), “stilbenoid, diarylheptanoid and gingerol biosynthesis” (three genes up regulated), “flavonoid biosynthesis” (four genes up regulated), “pentose phosphate pathway” (four genes up regulated and five down regulated), “fructose and mannose metabolism” (five genes up regulated), “terpenoid backbone biosynthesis” (six genes up regulated and three down regulated), “plant-pathogen interaction” (13 genes up regulated and two down regulated) and “phenylpropanoid biosynthesis” (seven genes up regulated and two down regulated) (Fig. 6A). In plant-pathogen interaction pathway, DEGs encoding RBOH (respiratory burst oxidase), CML (calcium-binding protein), MEKK1 (mitogen-activated protein kinase kinase kinase 1) were up regulated in the patchouliol-type, which could improve Cell wall reinforcement and plant defense response. The metabolic pathways related to carbohydrate and energy metabolism were “fructose and mannose metabolism” and “pentose phosphate pathway”, which played an important role in plant development. Otherwise, “stilbenoid, diarylheptanoid and gingerol biosynthesis”, “terpenoid backbone biosynthesis”, “ubiquinone and other terpenoid-quinone biosynthesis”, “sesquiterpenoid and triterpenoid biosynthesis”, “flavonoid biosynthesis” and “phenylpropanoid biosynthesis” were important secondary metabolism pathways. Among them “terpenoid backbone biosynthesis” and “sesquiterpenoid” were main ways to synthesize patchouli oil.

The KEGG pathways in the stems of the pogostone-type vs the patchouliol-type were mainly “brassinosteroid biosynthesis” with q value ≤2.00E−05 (five genes up regulated and two down regulated), “sesquiterpenoid and triterpenoid biosynthesis” q value ≤4.09E−02 (five genes up regulated and two down regulated), “flavonoid biosynthesis” (four genes up regulated and one down regulated), “phenylpropanoid biosynthesis” (nine genes up regulated and three down regulated), “ubiquinone and other terpenoid-quinone biosynthesis” (five genes up regulated), “stilbenoid, diarylheptanoid and gingerol biosynthesis” (three genes up regulated and one down regulated) and “plant hormone signal transduction” (13 genes up regulated and 11 down regulated) (Fig. 6B). The top 20 KEGG enrichment pathways of the stems were mainly composed of secondary metabolism and plant hormone signal transduction pathways. Among plant hormone signal transduction pathways, DEGs encoding JAR1 (jasmonic acid-amino synthetase 1), JAZ (Jasmonate ZIM domain-containing protein) and transcription factor MYC2 in Jasmonic acid (JA) biosynthesis and transduction pathway were up regulated in the patchouliol-type. And BR6OX2 (brassinosteroid-6-oxidase 2) in the BR downstream biosynthesis pathway (File S8) and GA2ox (gibberellin 2beta-dioxygenase) DEGs were up regulated in the patchouliol-type (File S9). However DEGs encoding ARF (auxin response factor) and SAUR family protein were down regulated in the auxin signal transduction of the patchouliol-type.

From KEGG pathway enrichment analysis, we found that the pathways of the leaves in the pogostone-type vs the patchouliol-type were mainly “diterpenoid biosynthesis” with q value ≤ 0.1852 (four genes up regulated and three down regulated), “phenylpropanoid biosynthesis” (17 genes up regulated and five down regulated), “sesquiterpenoid and triterpenoid biosynthesis” (five genes up regulated and two down regulated), “plant hormone signal transduction” (27 genes up regulated and 14 down regulated) and “plant-pathogen interaction” (27 genes up regulated and seven down regulated) (Fig. 6C). We found that the KEGG enrichment pathways of the leaves were mainly composed of secondary metabolism, plant hormone signal transduction and plant-pathogen interaction pathways. Among plant hormone signal transduction pathways, BSK (BR-signaling kinase), BIN2 (protein brassinosteroid insensitive 2), BZR1/2 (brassinosteroid resistant 1/2) in the brassinosteroid signal transduction pathway were expressed at higher levels in the patchouliol-type than in the pogostone-type (File S10). Furthermore, DEGs encoding JAR1 in the JA signal transduction pathway were up regulated in the patchouliol-type. DEGs encoding GA20ox (gibberellin-44 dioxygenase) and GA2ox in the diterpenoid biosynthesis pathway were up regulated in the patchouliol-type than in the pogostone-type (File S11). In plant-pathogen interaction pathway, DEGs encoding RBOH, CML, calmodulin (CALM), MEKK1 and FLS2 (LRR receptor-like serine/threonine-protein kinase) were up regulated and disease resistance protein RPM1 was down regulated in the patchouliol-type. Otherwise, the DEGs in the pathways of secondary metabolite biosynthesis indicated that many important ingredients, such as sesquiterpenes, triterpenoids, diterpenoids and flavonoids, were mainly synthesized in the leaves.

### Transcript profiles involved in terpenoid backbone biosynthesis

**Figure 7:**
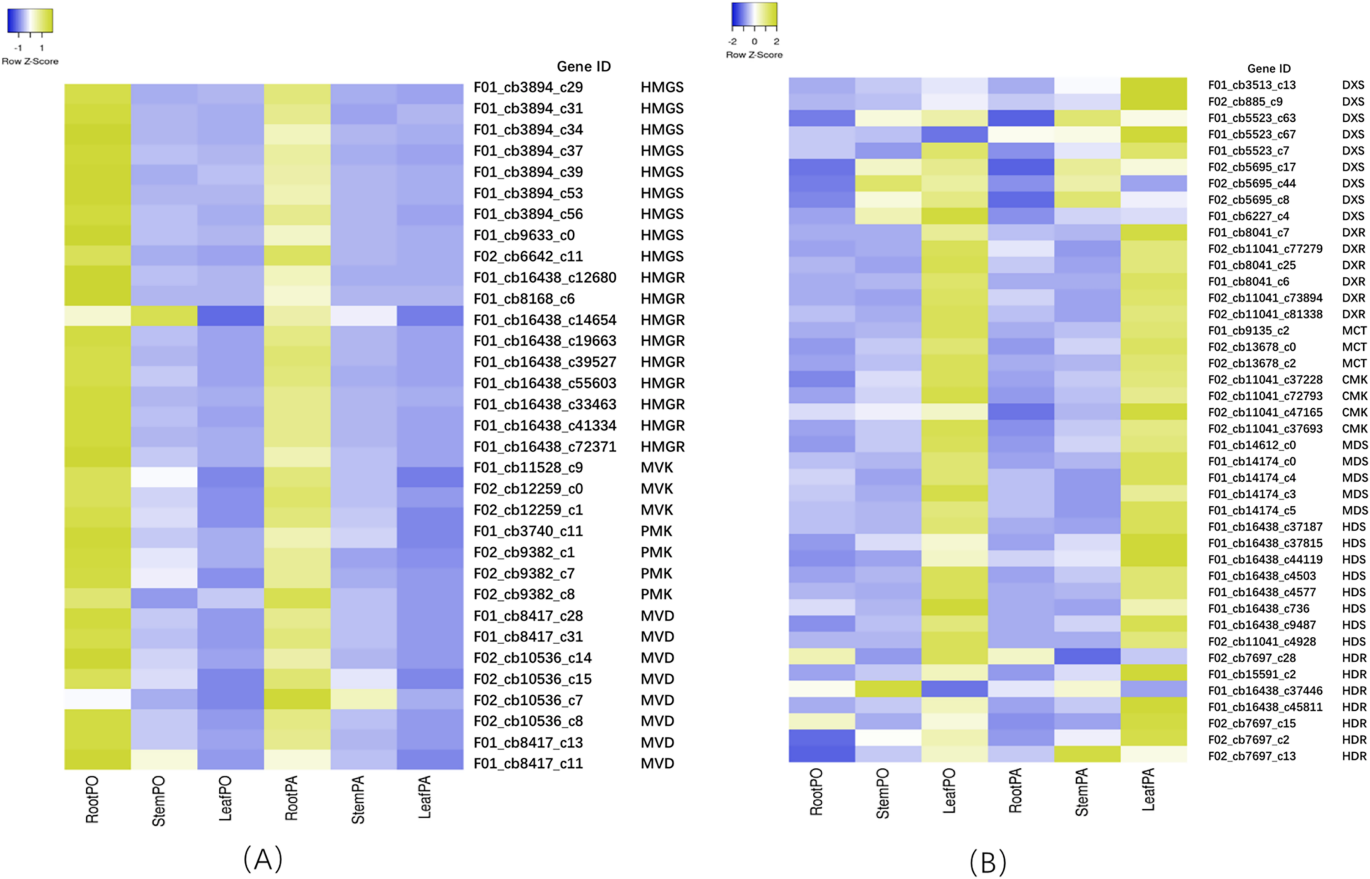
Heatmap of expression levels of the MVA and MEP pathway in root, stem and leaf of two chemical types. (A) DEGs of MVA related genes. (B) DEGs of MEP related genes. PA means the patchouliol-type and PO means the pogostone-type.

**Figure 8:**
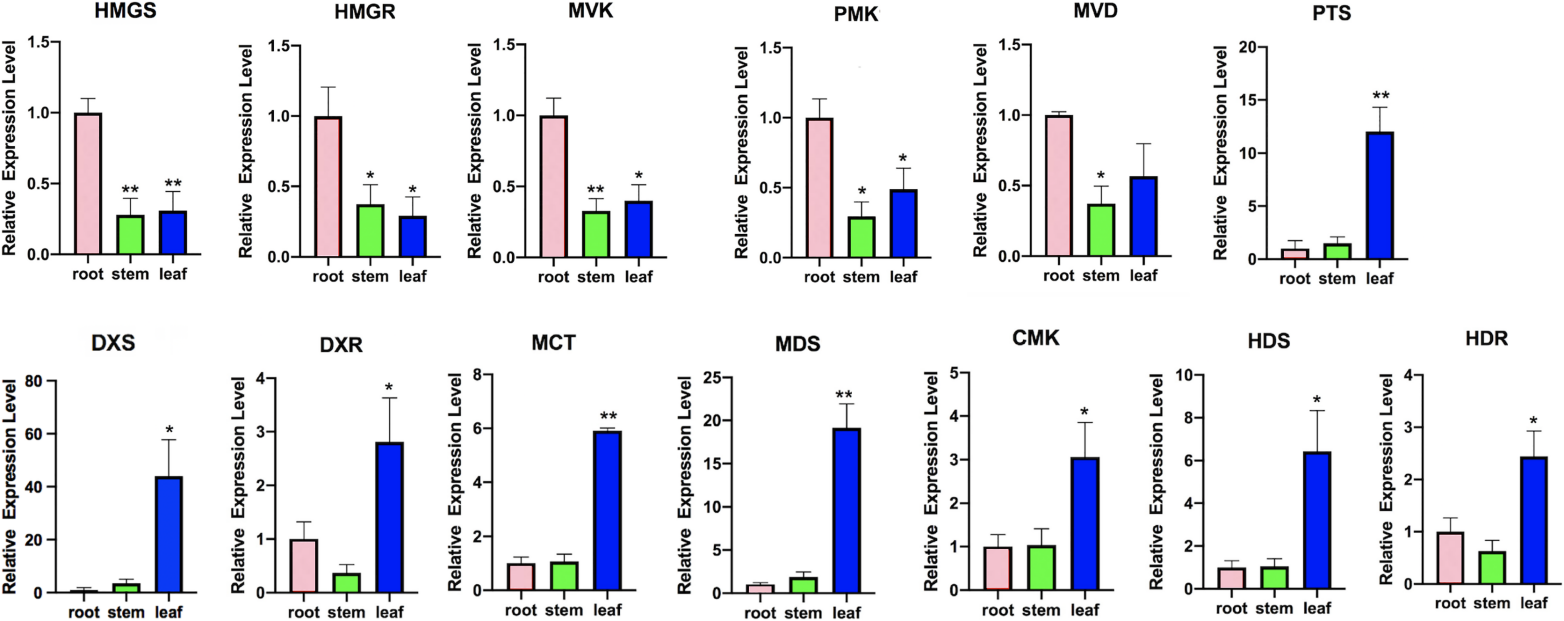
Expression analysis of transcripts involved in terpenoid backbone biosynthesis. Columns represent the value of relative expression levels. Error bars represent the SD from three independent experiments. The significance of the difference was analyzed using a one-way ANOVA (*P < 0.01, **P < 0.001).

To investigate the terpenoid backbone biosynthesis pathway in three tissues of *P. cablin*, we analyzed the transcripts in the terpenoid backbone biosynthesis pathway (Fig. 7 and File S12), and the expression patterns of the terpenoid backbone biosynthesis genes were detected by qRT-PCR (Fig. 8 and File S13).

In the MVA pathway, we found that most of the transcripts, such as hydroxy-3-methylglutaryl-CoA synthase (HMGS), 3-hydroxy-3-methylglutaryl-CoA reductase (HMGR), mevalonate kinase (MVK), phosphomevalonate kinase (PMK), and mevalonate diphosphate decarboxylase (MVD), showed higher expression levels in the roots but lower expression levels in the stems and leaves (Fig. 7A). However, in the MEP pathway, most of the transcripts, including 1-deoxy-D-xylulose-5-phosphate synthase (DXS), 1-deoxy-D-xylulose-5-phosphate reductoisomerase (DXR), 2-C-methyl-D-erythritol-4-phosphate cytidylyltransferase (MCT), 4-diphosphocytidyl-2-C-methyl-D-erythritol kinase (CMK), 2-C-methyl-D-erythritol 2,4-cyclodiphosphate synthase (MDS), 4-hydroxy-3-methylbut-2-en-1-yl diphosphate synthase (HDS), and 4-hydroxy-3-methylbut2-en-1-yl diphosphate reductase (HDR), showed higher expression levels in the leaves, with lower expression levels in the stems and the roots. Then, IPP and dimethylallyl pyrophosphate (DMAPP) are condensed from farnesyl diphosphate synthase (FPPS) to form the sesquiterpene (including patchouli alcohol) intermediate (Fig. 7B). In addition, we found that the expression of patchouli alcohol synthase (PTS) genes in the leaves was much higher than that in the roots and stems (Fig. 8).

## Discussion

### Evaluation of SMRT sequencing quality

In this study, the full-length transcriptome of *P. cablin* was sequenced using SMRT sequencing and NGS technology. In total, 574,817,420,978 ROIs, including 334,683 and 276,811 FLNCs, were obtained from the two cell lines. Second-generation transcriptome sequencing of 18 samples was completed, and a total of 125.83 G of clean data was obtained, ensuring that Q30 reached 85%. A total of 169,641, 152,493 consensus isoforms were obtained from F01 and F02, including 95,298 and 92,051 high-quality isoforms respectively. 134,647 nonredundant transcript isoforms were obtained using CD-HIT software. The average consensus isoform read lengths in F01 and F02 were 2,028 and 2,147 bp, respectively, which indicated that ROIs were long enough to represent full-length transcripts (Minoche et al., 2015; Tian et al., 2018).

Meanwhile, the second-generation data were used to correct the third-generation low-quality reads. Therefore, the resulting comprehensive transcriptome database for *P. cablin* could provide insight into the structures and functions of genes and the gene expression differences in the two chemical types.

### The role of the MEP pathway in terpenoid biosynthesis

There are two pathways for terpenoids biosynthesis in higher plants: the MVA pathway in the cytoplasm and the MEP pathway in plastids such as plant chloroplasts. However, increasing evidence indicates that the MVA and MEP pathways play different roles in terpenoids (Kasahara et al., 2002; Luo et al., 2003; Skorupinska-Tudek et al., 2008). In transgenic *Salvia miltiorrhiza* lines, DXS showed a much more powerful effect than HMGR in tanshinone biosynthesis, which indicated that genetic manipulation of the MEP pathway resulted in significantly higher yields than MVA pathway enhancement (Kai et al., 2011; Yang et al., 2012). The results suggested that the MEP pathway may play a major role in tanshinone biosynthesis. In our results, the expression levels of related genes in three vegetative tissues were very different in the two pathways. In the MVA pathway, the highest expression level of related biosynthetic genes was found in the roots, with the lower expression in the leaves and stems. However, in the MEP pathway, the highest expression level of related genes was found in the leaves, with the lower expression in the roots and stems. Targeting PTS into *Artemisia annua* plastid produced higher patchouli alcohol compared to its expression in cytoplasm (Fu et al., 2021). Because patchouli alcohol and sesquiterpene mainly synthesized in the leaves of *P. cablin*. Therefore, one of the explanations was that MEP pathway may probably play a much more important role in biosynthesis of patchouli alcohol and sesquiterpene. Another explanation was that because more chloroplasts (one kind of plastids) in leaves than roots or stems and the MEP pathway mainly took place in plastids, the gene expression level of the MEP pathway was higher in the leaves than the roots and the stems.

### DEG analysis of the two chemical types

From the results for the DEGs, the number of DEGs between two tissues of the same chemical type was greater than that in the same tissue of two chemical types. Regardless of the patchouliol-type or the pogostone-type patchouli, the number of DEGs between the roots and leaves was the largest, and the number of DEGs between the stems and the leaves was the smallest. Since each tissue has specific physiological functions and anatomical structures, the difference between the roots and leaves is usually greater than the difference between other plant vegetative tissues, so this pairing demonstrated the largest number of DEGs. The stems and leaves had similar physiological functions, such as photosynthesis, so the number of DEGs between them was minimal.

According to the number of DEGs in the same tissue of the two chemical types, leaves > stems > roots. The number of DEGs between the leaves of the two chemical types was approximately twice that of the roots. The data indicated that in addition to the differences in physiological functions, there may be more DEGs involved in the biosynthesis of secondary metabolites in the leaves, followed by the stems.

### Identification of hormone signaling-related genes in growth and development

Patchouliol-type patchouli usually has larger leaves, longer stems, higher stress resistance and more aromatic oil, while pogostone-type patchouli has smaller leaves, shorter stems, poor stress resistance and less aromatic oil.

JA and its derivatives are collectively named jasmonates (JAs). JAs are signaling molecules involved in the regulation of many physiological and developmental processes in plants (Lorenzo et al., 2004). In addition, JAs are important regulators in the plant response to biotic and abiotic stresses, such as ozone exposure, wounding, water deficit and pathogen/pest attack. Currently, JA, MeJA (a volatile methyl ester, methyl jasmonate) and JA-Ile (predominant amino acid conjugate, jasmonoyl-isoleucine) are known to function as signal molecules in plants (Li & Yan, 2014). Our previous study revealed that exogenous foliar spraying with MeJA could effectively increase the content of patchouli alcohol (He et al., 2014). DEG analysis found that JAR1 transcripts were upregulated in the leaves of patchouliol-type patchouli. JAR1 can conjugate Ile to JA-Ile, which binds to the receptor COI1 to activate most JA-elicited responses. The upregulation of the JAR1 DEGs may indicate more JA-elicited responses to induce patchouli alcohol and terpenoid synthesis.

Brassinosteroids (BRs) are a class of plant steroid hormones that play an extremely important role in plant growth and development and responses to several stresses, such as extreme temperatures and drought (Bai et al., 2012; Yang et al., 2011). C6 oxidation is a rate-limiting step in the BR biosynthesis pathway (Wei & Li, 2020), and the downstream enzyme BR6OX2 is a rate-limiting enzyme(Gruszka, Szarejko & Maluszynski, 2011). Loss of either BR6OX2 or BR6OX2 farnesylation could result in reduced brassinolide accumulation (Jamshed et al., 2017). The upregulation of BR6OX2 DEGs possibly resulted in the synthesis of more BRs (Wei et al., 2017).

Gibberellins (GAs) are plant hormones that are essential for many developmental processes in plants, including seed germination, stem elongation, leaf expansion, and trichome development (Achard & Genschik, 2009). The major bioactive GAs, which include GA1, GA3, GA4 and GA7, are derived from a basic diterpenoid carboxylic acid skeleton (Yamaguchi, 2008). According to the KEGG enrichment analysis between two chemical types, the GA20ox and GA2ox transcripts were upregulated in the diterpenoid biosynthesis pathway of the patchouliol-type. GA20ox and GA2ox play key roles in the entire process of GA metabolism. They are responsible for the last several steps in the synthesis of active GAs, whereas GA2ox members catalyze the inactivation of bioactive GAs (Shang et al., 2017). The higher GA20ox and GA2ox gene expression levels suggest that more GAs may be synthesized in the patchouliol-type than in the pogostone-type. From the expression of related genes in the plant hormone synthesis pathways, we found that JAR1, BR6OX2, GA20ox and GA2ox DEGs were all upregulated in the patchouliol-type. In this case, more JAs, GAs and BRs could be synthesized in the tissues of the patchouliol-type. Therefore, these plant hormones could promote the growth and resistance of patchouliol-type patchouli.

## Conclusions

The two chemical types of patchouli exhibit many differences in morphology and chemical compositions. Our work demonstrated a comprehensive view of the molecular mechanisms of morphology and terpenoid biosynthesis by using a combination of SMRT and next-generation sequencing. Hormone levels may play an important role in the formation of two chemical types. These findings help to understand the formation mechanisms of the two chemical types and terpenoid biosynthesis in *P. cablin*.

## Supplemental Information

10.7717/peerj.12940/supp-1 Supplemental Information 1 Primers used in qRT-PCR.

10.7717/peerj.12940/supp-2 Supplemental Information 2 Integrated function annotation.

10.7717/peerj.12940/supp-3 Supplemental Information 3 The genes related to terpenoid biosynthesis.

10.7717/peerj.12940/supp-4 Supplemental Information 4 IncRNA predict combine.

10.7717/peerj.12940/supp-5 Supplemental Information 5 TF classification.

10.7717/peerj.12940/supp-6 Supplemental Information 6 DEG comparison of two chemical types.

10.7717/peerj.12940/supp-7 Supplemental Information 7 Statistical table of the number of differentially expressed transcripts.

10.7717/peerj.12940/supp-8 Supplemental Information 8 Brassinosteroid biosynthesis pathway of stem DEGs.

10.7717/peerj.12940/supp-9 Supplemental Information 9 Diterpenoid biosynthesis of stem DEGs.

10.7717/peerj.12940/supp-10 Supplemental Information 10 Diterpenoid biosynthesis of leaf DEGs.

10.7717/peerj.12940/supp-11 Supplemental Information 11 Plant hormone signal transduction of leaf DEGs.

10.7717/peerj.12940/supp-12 Supplemental Information 12 Expression_levels_of_the_MVA_and_MEP_pathway_in_root,_stem_and_leaf_of_two_chemical_types.

10.7717/peerj.12940/supp-13 Supplemental Information 13 Gene qRT-PCR verify.

## Additional Information and Declarations

### Data Availability

The following information was supplied regarding data availability: The data is available at Genbank: PRJNA607886, PRJNA606892.
