# Supplementary material for: SMRT sequencing of full-length transcriptome and gene expression analysis in two chemical types of Pogostemon cablin (Blanco) Benth
Source: PeerJ. 2022 Feb 22;10:e12940. doi: 10.7717/peerj.12940 (PMC8877398; doi:10.7717/peerj.12940)
Supplement: Supplemental Information 7 [file peerj-10-12940-s007.docx]

Table S7 Statistical table of the number of differentially expressed transcripts.

| **DEG Set** | **All DEGs** | **upregulated** | **downregulated** |
| --- | --- | --- | --- |
| Root_PO__vs_Stem_PO_ | 14990 | 8225 | 6765 |
| Root_PO_ _vs_Leaf_PO_ | 27108 | 14893 | 12215 |
| Root_PO_ _vs_ Root_PA_ | 1352 | 851 | 501 |
| Stem_PO_ _vs_ Leaf_PO_ | 20377 | 10730 | 9647 |
| Stem_PO_ _vs_ Stem_PA_ | 1752 | 1072 | 680 |
| Leaf_PO_ _vs_ Leaf_PA_ | 2738 | 1833 | 905 |
| Root_PA_ _vs_ Stem_PA_ | 12344 | 6249 | 6095 |
| Root_PA_ _vs_ Leaf_PA_ | 23660 | 13295 | 10365 |
| Stem_PA_ _vs_ Leaf_PA_ | 12379 | 7868 | 4511 |

DET Set: the name of the differentially expressed transcript set;

All DEGs: the number of differentially expressed transcripts;

upregulated: the number of upregulated transcripts;

downregulated: the number of downregulated transcripts.

PO：the pogostone-type

PA：the patchouliol-type
