# Supplementary figures and images for: SMRT sequencing of full-length transcriptome and gene expression analysis in two chemical types of Pogostemon cablin (Blanco) Benth
Source: PeerJ. 2022 Feb 22;10:e12940. doi: 10.7717/peerj.12940 (PMC8877398; doi:10.7717/peerj.12940)

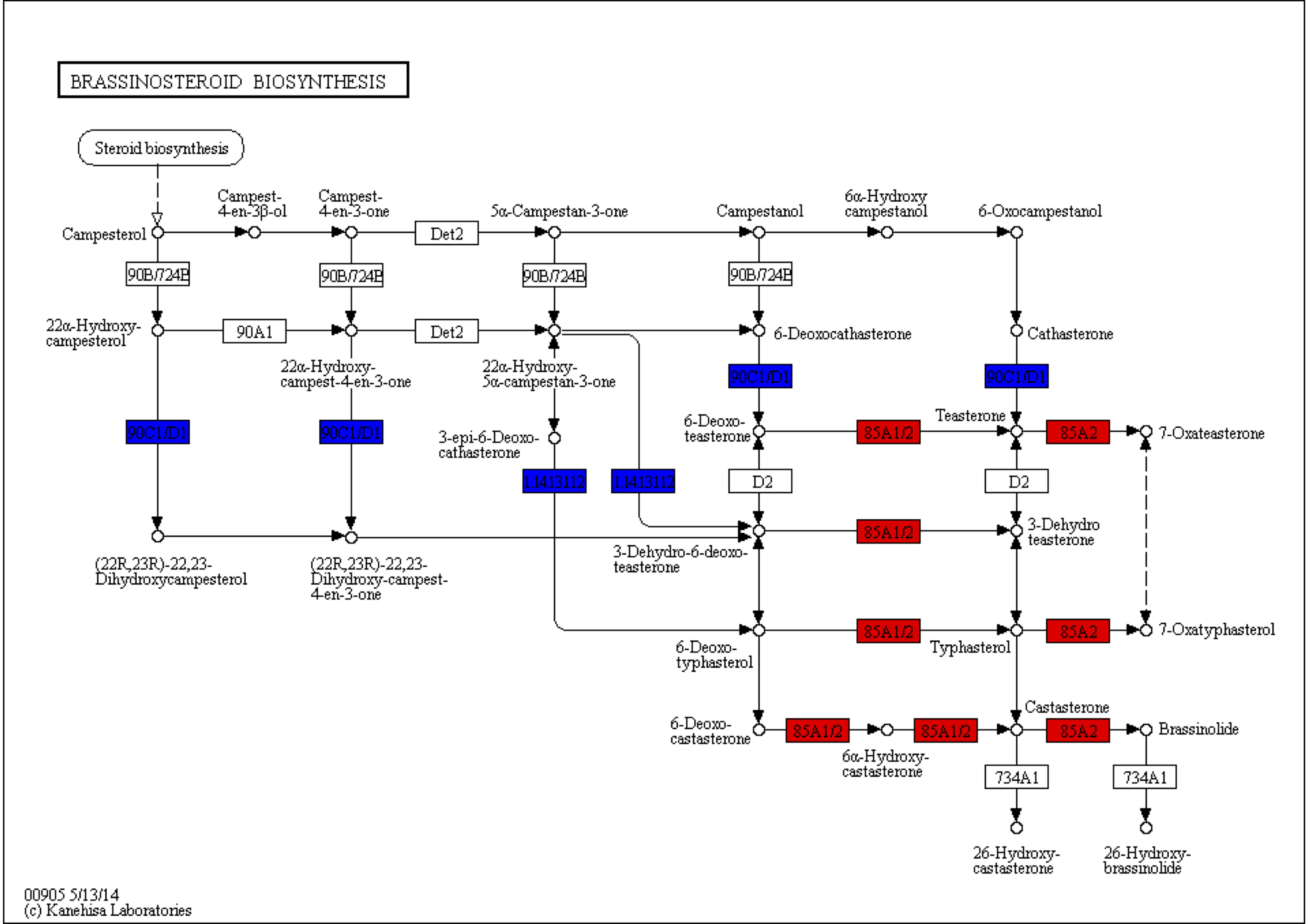

Supplement: Supplemental Information 8 [file peerj-10-12940-s008.png]

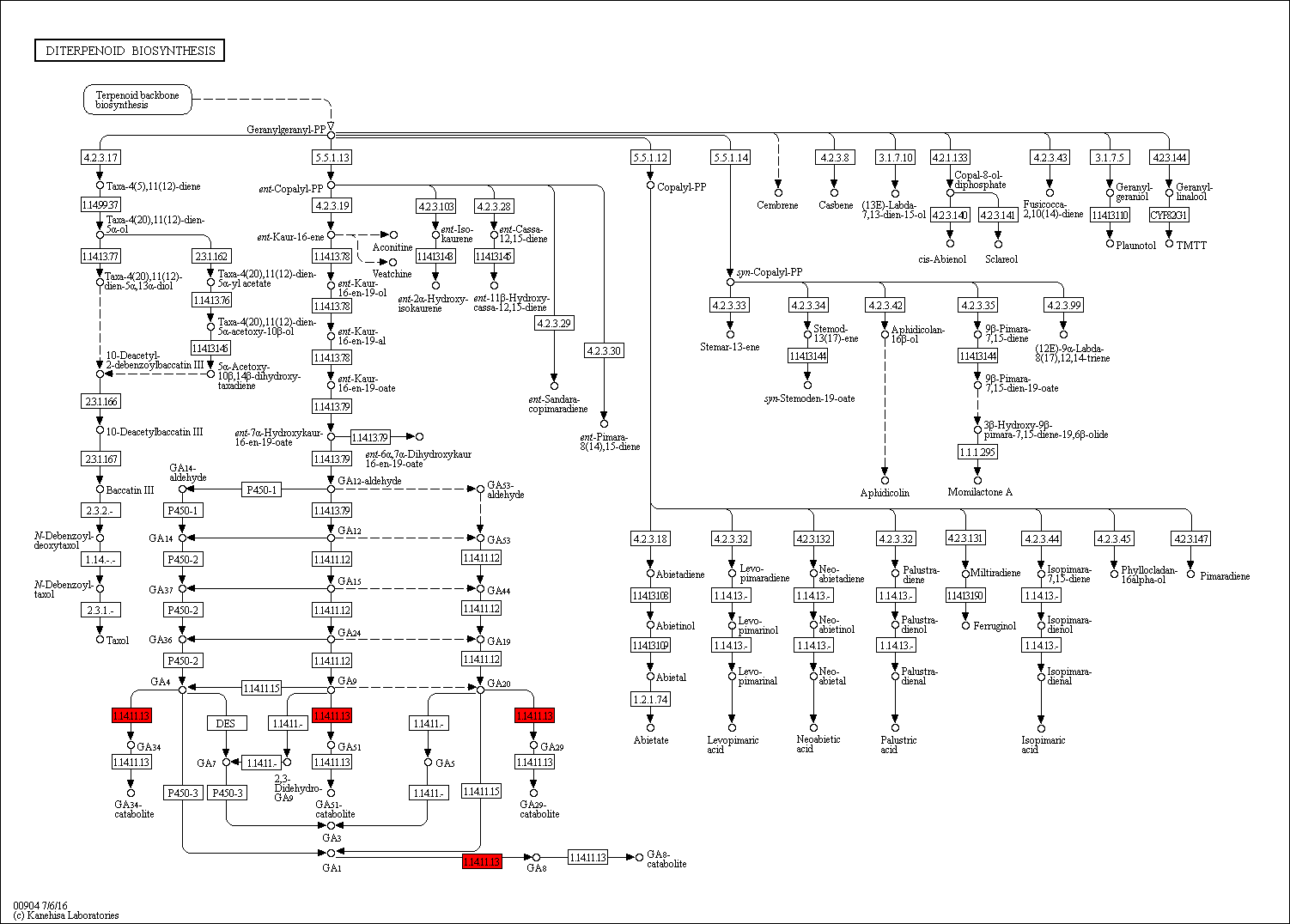

Supplement: Supplemental Information 9 [file peerj-10-12940-s009.png]

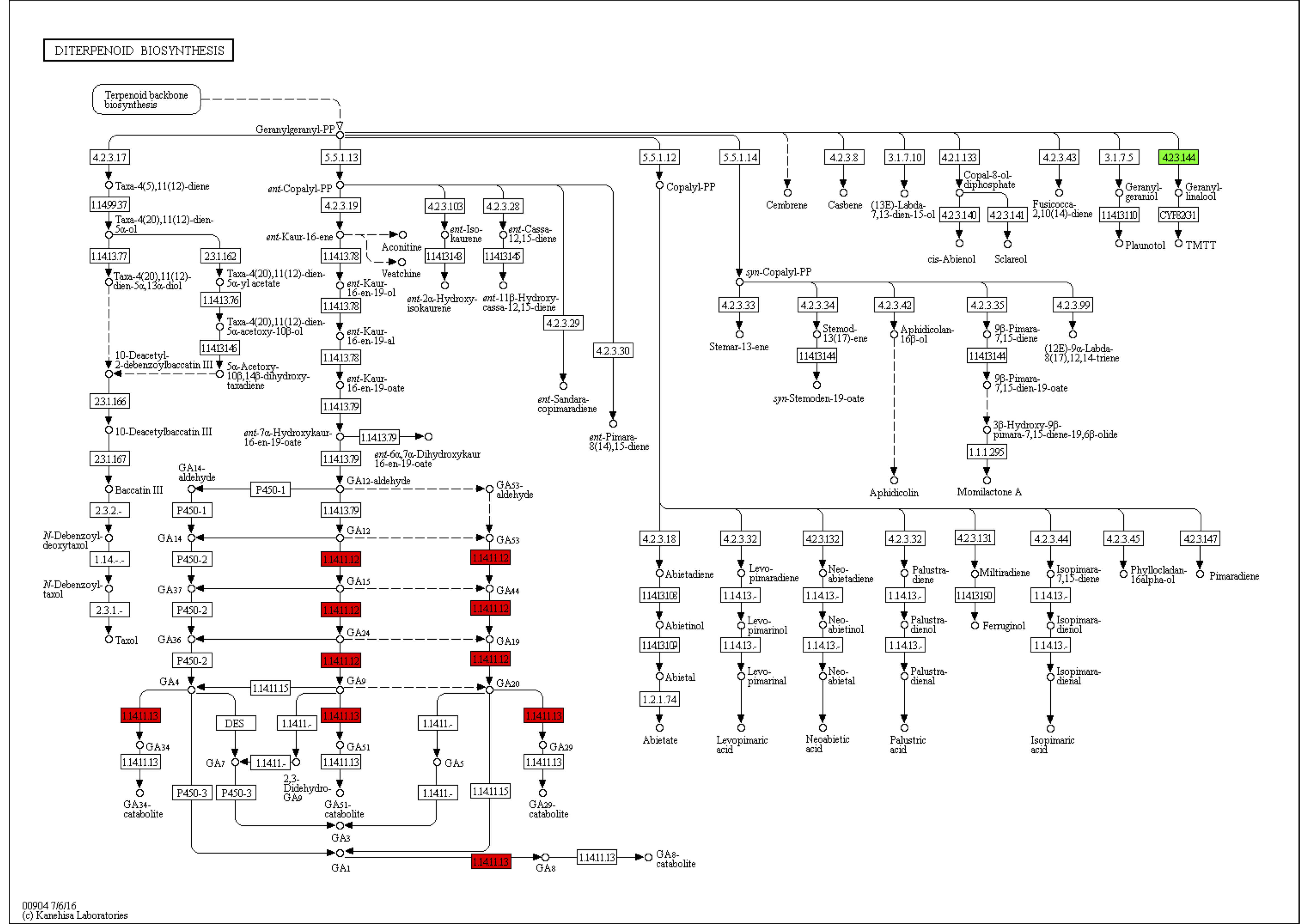

Supplement: Supplemental Information 10 [file peerj-10-12940-s010.png]

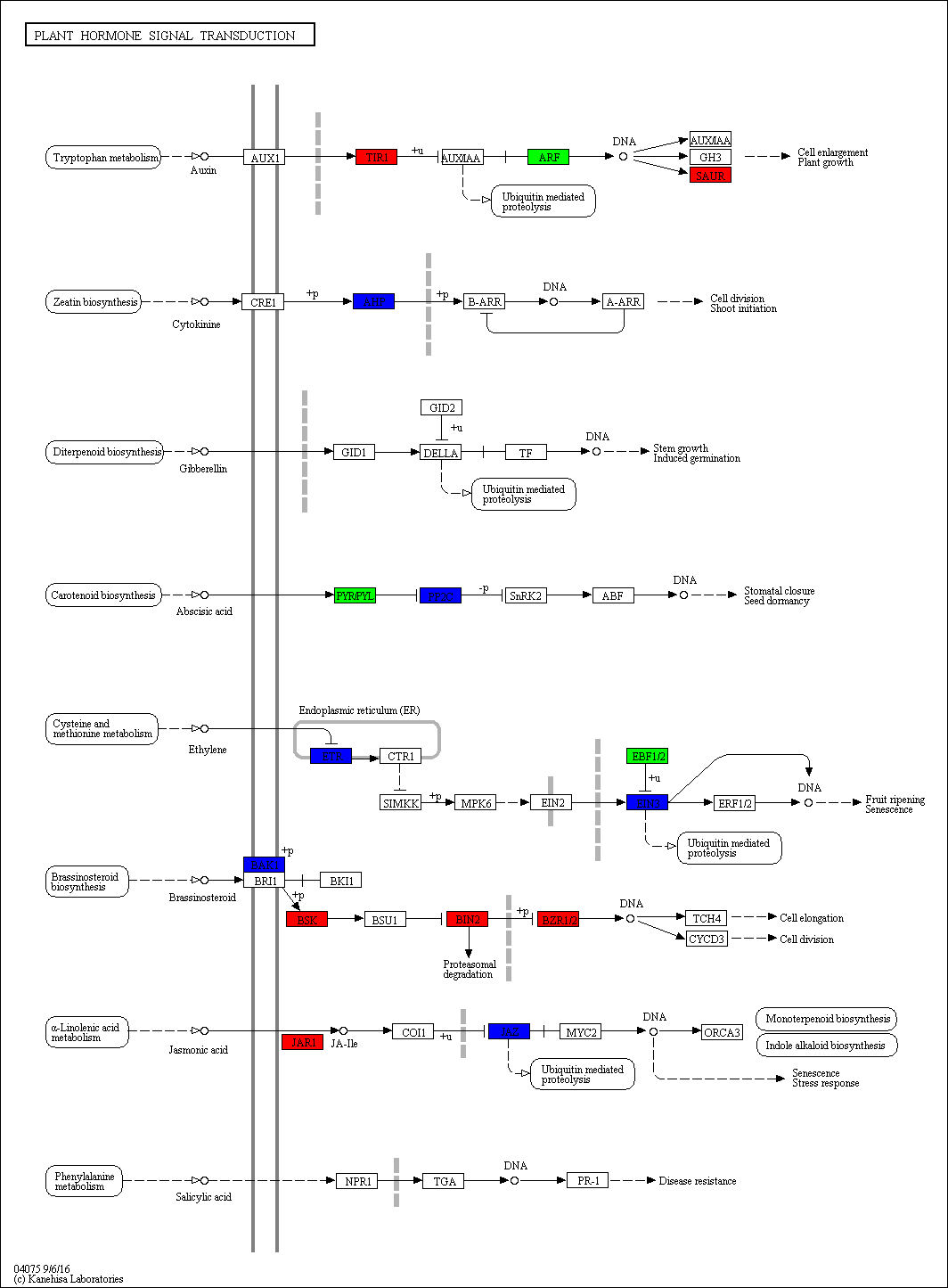

Supplement: Supplemental Information 11 [file peerj-10-12940-s011.png]
